# Modulation of Multidrug Resistance Gene Expression by Coumarin Derivatives in Human Leukemic Cells

**DOI:** 10.1155/2017/5647281

**Published:** 2017-12-13

**Authors:** Tomasz Kubrak, Anna Bogucka-Kocka, Łukasz Komsta, Daniel Załuski, Jacek Bogucki, Dariusz Galkowski, Robert Kaczmarczyk, Marcin Feldo, Maria Cioch, Janusz Kocki

**Affiliations:** ^1^Centre for Innovative Research in Medical and Natural Sciences, Faculty of Medicine, University of Rzeszow, Rzeszow, Poland; ^2^Department of Biology and Genetics, Medical University of Lublin, Lublin, Poland; ^3^Department of Medicinal Chemistry, Medical University of Lublin, Lublin, Poland; ^4^Department of Pharmacognosy, Ludwik Rydygier Collegium Medicum, Nicolaus Copernicus University, 9 Marie Curie-Skłodowska Street, 85-094 Bydgoszcz, Poland; ^5^Warsaw Higher Humanistic School, Warsaw, Poland; ^6^Praesum Healthcare Services, Lake Worth, FL 33461, USA; ^7^Department of Neurosurgery and Paediatric Neurosurgery, Medical University of Lublin, Lublin, Poland; ^8^Department of Vascular Surgery and Angiology, Medical University of Lublin, Lublin, Poland; ^9^Department of Hemato-Oncology and Bone Marrow Transplantation, Medical University of Lublin, Lublin, Poland; ^10^Department of Clinical Genetics, Medical University of Lublin, Lublin, Poland

## Abstract

The presence of multidrug resistance (MDR) in tumor cells is considered as the major cause of failure of cancer chemotherapy. The mechanism responsible for the phenomenon of multidrug resistance is explained, among others, as overexpression of membrane transporters primarily from the ABC family which actively remove cytostatics from the tumor cell. The effect of 20 coumarin derivatives on the cytotoxicity and expression of *MDR1*, *MRP1*, *BCRP*, and *LRP* genes (encoding proteins responsible for multidrug resistance) in cancer cells was analyzed in the study. The aim of this research included determination of IC10 and IC50 values of selected coumarin derivatives in the presence and absence of mitoxantrone in leukemia cells and analysis of changes in the expression of genes involved in multidrug resistance: *MDR1*, *MRP*, *LRP*, and *BCRP* after 24-hour exposure of the investigated cell lines to selected coumarins in the presence and absence of mitoxantrone in IC10 and IC50 concentrations. The designed research was conducted on 5 cell lines derived from the human hematopoietic system: CCRF/CEM, CEM/C1, HL-60, HL-60/MX1, and HL-60/MX2. Cell lines CEM/C1, HL-60/MX1, and HL-60/MX2 exhibit a multidrug resistance phenotype.

## 1. Introduction

Compounds of natural origin and their derivatives play an increasingly important role in medicine and pharmacology. Approximately 60% of therapeutic drugs used in the treatment of cancer are compositions comprising natural compounds and/or their derivatives [1]. The main problem of cancer chemotherapy is the adverse effects resulting in high cytotoxicity toward normal rapidly proliferating cells, especially the bone marrow and gastrointestinal tract. In order to mitigate the side effects, modified therapeutic regimens such as combination therapy have been introduced [2–4]. Several hundred membrane transporters in two major protein superfamilies ATP-binding cassette (ABC) and solute carrier (SLC) can be found in humans. The transporters may represent the rate determining step in pharmacokinetics and drug-drug interactions [5, 6]. ABC transporters, among other functions, use the energy of ATP binding and hydrolysis to actively transport chemicals across extra- and intracellular membranes.

Subfamilies of multidrug resistance proteins (MDRs and ABCB), multidrug resistance-associated proteins (MRPs and ABCC), and breast cancer resistance protein (BCRP and ABCG2) also belong to the human ABC transporter family [5].

The phenomenon of multidrug resistance caused by overexpression of these ABC drug transporters in cancer cells confers cross-resistance to a multitude of drugs and presents a significant obstacle limiting the effectiveness of cancer chemotherapy. In recent years, a number of natural, plant-derived compounds have been found to inhibit proliferation, induce apoptosis, suppress angiogenesis, retard metastasis, and enhance chemotherapy exhibiting anticancer potential both in vitro and in vivo. Many researchers point to the use of natural products as inhibitors of multidrug resistance and often call them “fourth generation modulators” [7, 8].

The occurrence of multidrug resistance was first described by Biedler and Riehm in 1970 during incubation of leukemia cells from a *Syrian hamster* and mice in an increasing concentration of actinomycin D. They encountered not only resistance to this particular drug but also to many others including daunorubicin and vinblastine [9]. However, the real breakthrough occurred in 1976 when Juliano and Ling described for the first time the now classical P-glycoprotein (ABCB; P-gp), which is the first known human protein responsible for the occurrence of the multidrug resistance [10]. Numerous studies showed a close relationship between overexpression of P-gp and a lower rate of cancer remission with a higher incidence of resistance to treatment. This observation underlines the importance of the mechanism of multidrug resistance-related P-gp in cancer. In addition, some studies provided evidence that expression of P-gp may be a factor the clinical outcome of therapy in certain tumors such as breast cancer and neuroblastoma or sarcoma in children [11, 12]. Based on these observations and findings, we can state that the future success of anticancer therapy is insignificant degree dependent on the results of research targeted to overcome multidrug resistance [13–15]. Mitoxantrone is a synthetic anthracenedione that has been used in the clinical treatment of various cancers. The anticancer mechanisms of mitoxantrone are believed to be related to its capacity to bind DNA and inhibit DNA topoisomerase II in the nuclear compartment of cells. In addition, the action of its metabolites in the intracellular cytosolic compartment may also contribute to the antineoplastic activities of mitoxantrone [16, 17].

It was reported that plant-derived polyphenolic compounds, mainly flavonoids and stilbenes or their synthetic derivatives, can modulate the main ABC transporters responsible for cancer drug resistance, including P-gp, multidrug resistance-associated protein 1 (MRP1), and breast cancer resistance protein (BCRP) [18]. The coumarins are secondary plant metabolites that are characterized by enormous structural diversity. They have very diverse mechanisms of action. Their biological activity is determined by their lactone structure, whereas pharmacological properties are determined by the structure of compounds [19].

Some of the coumarins, such as aesculetin, aesculin, and fraxin, also possess antioxidant activity. It was confirmed that acute lymphocytic leukemia (ALL) and acute nonlymphocytic leukemia (ANLL) have increased levels of various reactive oxygen (ROS) such as superoxide radicals, H_2_O_2_, and decreased levels of enzymatic (SOD and CAT) and nonenzymatic antioxidants compared to healthy individuals [20–22].

There are many publications about relationships that modulate multidrug resistance that are not used in the clinic due to weak action or side effects. There is also a need to look for substances that overcome the drug resistance phenomenon of tumor cells. Therefore, testing effective compounds such as coumarins which can reverse drug resistance is warranted [23].

In our previous papers, coumarin derivatives were screened for their cytotoxic activity against human tumor cells and several were found to exhibit potent cytotoxic activity [24–31]. These studies led to this analysis of the impact of coumarin derivatives to reverse drug resistance in five human leukemic cell lines via multidrug resistance genes expression. In a continuing search for potent and selective cytotoxic coumarin derivatives as antitumor agents, we analyzed 20 coumarin derivatives and evaluated their cytotoxic effects against human leukemic cells and the impact on *MDR1*, *MRP1*, *BCRP*, and *LRP* gene expression.

## 2. Materials and Methods

### 2.1. Cell Lines and Cell Culture

Human acute promyelocytic leukemia cell lines HL60, HL60/MX1, and HL60/MX2 and acute lymphoblastic leukemia cell lines CEM/C1 and CCRF/CEM were used. Cell lines were obtained from the American Type Culture Collection (ATCC) 10801, University Boulevard Manassas, VA 20110, USA. HL-60 (CCL 240) is a promyelocytic cell line derived by Collins (1987). The peripheral blood leukocytes were obtained by leukopheresis from a 36-year-old Caucasian female with acute promyelocytic leukemia. HL-60/MX1 (CRL–2258), a mitoxantrone-resistant derivative of the HL-60 cell line, was obtained from peripheral blood leukocytes obtained by leukopheresis from a patient with acute promyelocytic leukemia. HL-60/MX2 (CRL–2257) is also a mitoxantrone resistant derivative of the HL-60 cell line. HL-60/MX2 cells display atypical multidrug resistance (MDR) with the absence of P-gp overexpression and altered topoisomerase II catalytic activity and reduced levels of topoisomerase II alpha and beta proteins. CCRF/CEM (CCL–119) was derived from human lymphoblasts from the peripheral blood of a child with acute leukemia. CEM/C1 is a camptothecin- (CPT-) resistant derivative of the human T cell leukemia cell line CCRF/CEM. The cell line was selected and subcloned in 1991 for resistance to CPT (http://www.lgcstandarts-atcc.org/). The cells were maintained in RPMI 1640 medium (PAA Laboratories, Linz, Austria) supplemented with 10% fetal bovine serum (FBS) (PAA Laboratories) for HL60/MX1, HL60/MX2, CEM/C1, and CCRF/CEM cell lines and 20% FBS for HL60 cells, penicillin-streptomycin (100 U/mL PAA Laboratories), and amphotericin (PAA Laboratories) at 37°C in a humidified atmosphere of 5% CO_2_.

### 2.2. Analysis of Cell Viability

Cells were seeded on 12-well plates (Sarstedt, Wiener. Neudorf, Austria) at an initial density of 1 × 10^6^ cells/ml. After 24 hours, the cell suspension was stimulated with coumarin derivatives at concentrations ranging from 10 *μ*M to 1000 *μ*M. After 24 hours, 1 mL of cell suspension was centrifuged at 1000 rpm for 5 minutes and the supernatant was discarded. The cells were resuspended in 50 *μ*L PBS. From each tube, a 10 *μ*L cell suspension was taken and mixed with 10 *μ*L of Trypan blue reagent (Bio-Rad, Hercules, CA, USA). The sample was incubated for 5 minutes. Cell viability was measured by TC20 Automated Cell Counter (Bio-Rad). Each experiment was repeated three times.

### 2.3. Standards and Reagents

Isopimpinellin (ISO), bergapten (BER), xanthotoxol (XOL), xanthotoxin (XIN), byakangelicin (BIN), byakangelicol (BOL), heraclenin (HEC), phellopterin (FEL), herniarine (HER), aesculetin (AET), dihydrocoumarin (DHD), coumarin (COU), aesculin (AEL), umbelliferone (UMB), 4-methylo-7-methoxycoumarin (4,7M), 4-methylo-7-ethoxycoumarin (4,7E), 7-methylocoumarin (7ME), 6-methylocoumarin (6ME), 0,0-dimethylofraxetin (OOD), and scoparone (SCO) were purchased from ChromaDex® (ChromaDex, Irvine, CA, USA).

### 2.4. Determination of Gene Expression

Relative gene expression of *MDR1*, *MRP1*, *BCRP*, and *LRP* was assessed by real-time quantitative PCR and 2^−ΔΔC^_T_ method. Genes were quantitatively assessed in each sample taken from the research group and referred to gene expression determined in the corresponding samples in the control group 1 : 1.

#### 2.4.1. Cell Preparation

Cells were seeded on 12-well plates (Sarstedt, Wiener. Neudorf, Austria) at an initial density of 1 × 10^6^ cells/ml. After 24 hours, the cell suspension was stimulated with coumarin derivatives separately at IC10 and IC50 concentrations. Another group of cells was stimulated with coumarin derivative sat IC10 and IC50 concentration with mitoxantrone (+M) at a concentration of 0.02 *μ*M. We used two controls—cell cultures without stimulators and cell cultures with mitoxantrone at a concentration of 0.02 *μ*M. After 24 hours, the cell suspension (from each well) was centrifuged at 800 rpm for 5 minutes, and the supernatant was discarded.

#### 2.4.2. Isolation of Total Cellular RNA

To isolate total cellular RNA, we followed the method of Kocki et al. with modification, using a TRI-Reagent Solution (Ambion, USA) [32]. During this process, samples of cells were mixed with 250 *μ*l TRI-Reagent buffer (Ambion, USA) to obtain a homogenous suspension. Samples were then incubated for 5 min at room temperature until complete dissociation. At the next stage, 50 *μ*l chloroform (Sigma-Aldrich, USA) was added to the sample and shaken for 15 s. Next, the samples were left for 15 min to incubate at room temperature after which they were centrifuged for 15 min at 14,000 rpm at 4°C in 5415R Eppendorf centrifuges. The aqueous phase was placed in a new tube and 250 *μ*l 2-propanol (Sigma-Aldrich, USA) was added. The samples were thoroughly mixed and incubated for 20 min at room temperature. Following that, the mixtures were centrifuged for 20 min at 14,000 rpm at 4°C in 5415R Eppendorf centrifuges. Aqueous phase was removed from the above precipitate. The RNA precipitate was washed in cool 80% ethanol and obtained RNA samples were stored in 80% ethanol at −80°C for further analysis.

#### 2.4.3. Quantitative and Qualitative Analysis of RNA

The RNA concentration and purity were measured by spectrophotometry on a NanoDrop2000 (Thermo Scientific, USA). Precipitate of RNA in 80% ethanol was taken out at −20°C and next centrifuged for 15 min at 14,000 rpm at 4°C in 5415R Eppendorf centrifuges. The liquid part was removed, and RNA pellets were left to dry at room temperature. Subsequently, the precipitate was dissolved in DNase-, RNase-, and protease-free water (Sigma-Aldrich, USA) at 4°C, the volume depending on RNA concentration.

#### 2.4.4. cDNA Synthesis

The cDNA was synthesized using High-Capacity cDNA Reverse Transcription Kit, according to manufacturer's instructions [33]. Each reactive mixture contained the following set of reagents: 1 × RT buffer, 20 U RNase inhibitor, 50 U reverse transcriptase (MultiScribe Reverse Transcriptase), 1 × RT random primers, and 4 mM of each deoxynucleotide: dATP, dGTP, dTTP, and dCTP plus examined 1 *μ*g RNA in DNase-, RNase-, and protease-free water (Sigma-Aldrich, USA) to complete the volume required for reaction. Final volume of the reactive mixture was 20 *μ*l. Afterwards, the reactive components were thoroughly mixed and centrifuged to fuse them well. The cDNA was synthesized on Veriti Dx (Applied Biosystems, USA) under the following conditions: stage I, 25°C (10 min); stage II, 37°C (120 min); stage III, 85°C (5 min); and stage IV, 4°C.

#### 2.4.5. The qPCR Protocol

The cDNA, which was obtained by reverse transcription (RT) procedure, was amplified by real-time gene expression analysis (qPCR) on a 7900HT Real-Time Fast System [33], using the manufacturer's SDS software. Triplicate qPCR reactions were conducted for each sample. To exclude reagent contamination by foreign DNA, a blind trial was always performed without a DNA target. Reaction components included 11.25 *μ*l mixture of cDNA probe and 1.25 *μ*l oligonucleotide starters specific for genes examined and 12.5 *μ*l buffer TaqMan Universal PCR Master Mix. The reaction was performed on an optic reaction plate in required reactive volume 25 *μ*l, using probe sets of TaqMan Gene Expression Assays [33] with FAM-NFQ markers and oligonucleotide starters for human genes *MDR1*, *MRP1*, *BCRP*, and *LRP* and the housekeeping gene *GAPDH* was used as an internal control gene. Amplification protocol included in the following cycles: initial denaturation: 95°C, 10 min; and 40 cycles each composed of two temperatures: 95°C, 15 s and 60°C, 1 min. The number of copies of DNA molecules was monitored and calculated on a 7900HT Real-Time Fast System [33] in each amplification cycle. To calculate the number of examined DNA molecules present in the mixture at the onset of reaction, the number of PCR cycles after which the level of fluorescence exceeded the defined threshold cycle (CT) RQ Study Software [33] was used. The CT value for each sample of the endogenous control gene (*GAPDH*) was used to normalize the level of the interesting gene expression. The relative level of gene expression was calculated according to [34].

The RQ defines the expression of an examined gene in a stimulated cell sample with reference to the gene expression in the control cell sample calibrator (without stimulation). Finally, the RQs were analyzed after their logarithmic conversion into logarithm of RQ (LogRQ) [33]. Thus, the obtained results were more legible. LogRQ takes value greater, equal to or less than zero. LogRQ = O means that gene expression in the calibrated sample and the stimulated one are the same. LogRQ < 0 points to decreased gene expression in the stimulated cell sample, whereas LogRQ > 0 points to signal increased gene expression in the stimulated cell sample compared to the calibrated one.

#### 2.4.6. Statistical Analysis

The results obtained for stem cells were statistically analyzed by STATISTICA software by means of the nonparametric Mann–Whitney *U* test, Spearman rho correlation analysis, and Kruskal–Wallis test. The results obtained for cell lines were analyzed by chemometric techniques: cluster analysis based on Euclidean distance and Parallel Factor Analysis (PARAFAC). Data were presented as means ± SEM. The level of statistical significance was set at *p* < 0.05.

## 3. Results

### 3.1. Analysis of Cytotoxicity

The cytotoxicity of the examined coumarins was estimated using trypan blue vital staining in the presence of mitoxantrone M(+) and absence of mitoxantrone. The experiment was performed in triplicate and the mean values were calculated from the given values (Tables 1, 2, 3, and 4).

The cells of the 5 cancer cell lines exposed to coumarin derivatives presented diverse cytotoxicity dependent on the dose of IC10, IC10M(+), IC50, and IC50 M(+).

Cytotoxicity depends on both the type of relationship and the type of cell line. It turned out that the most sensitive cell lines to the IC10 dose were CCRF/CEM > HL-60 > CEM/C1 > HL-60/MX2 > HL-60/MX1 and to the IC50 dose without mitoxantrone were CCRF/CEM > CEM/C1 > HL-60 > HL-60/MX1 > HL-60/MX2.

It appeared that the most sensitive cell lines to the IC10 dose were CCRF/CEM > HL-60 > CEM/C1 > HL-60/MX2 > HL-60/MX1 and to the IC50 dose with mitoxantrone were CEM/C1 > CCRF/CEM > HL-60 > HL-60/MX2 > HL-60/MX1, respectively.

### 3.2. Analysis of Gene Expression Using Chemometry

In a preliminary statistical analysis of data results, descriptive statistics, minimal and maximal values, and means and standard variances were used.

To investigate a similarity between the behavior of investigated compounds, cell lines, and gene expression changes connected with multidrug resistance after exposition to investigated coumarin derivatives, a dimensionality reduction was applied using the chemometric techniques: cluster analysis and Parallel Factor Analysis (PARAFAC).

To investigate a distribution of similarities of coumarin derivative action, a total chemometric analysis (of all expression change values) was also performed.

**Figure 1:**
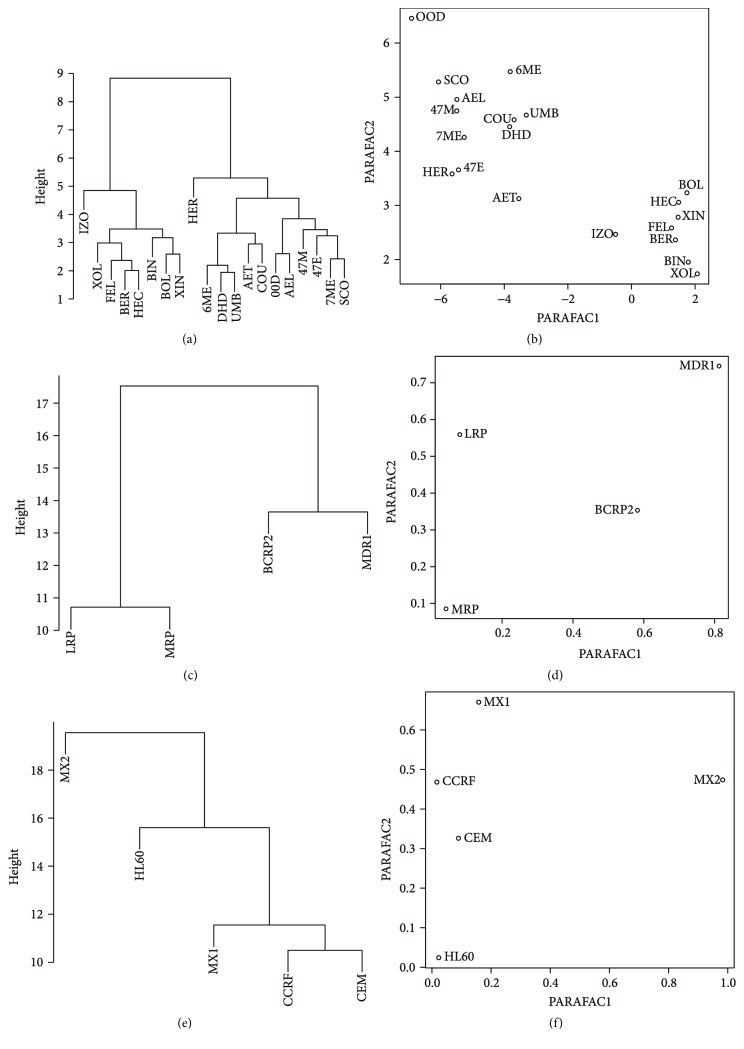
Comparison of similarities between coumarin derivatives (a, b), genes (c, d) and cell lines (e, f) by cluster analysis with Euclidean distance (a, c, e) and PARAFAC (b, d, f).

**Figure 2:**
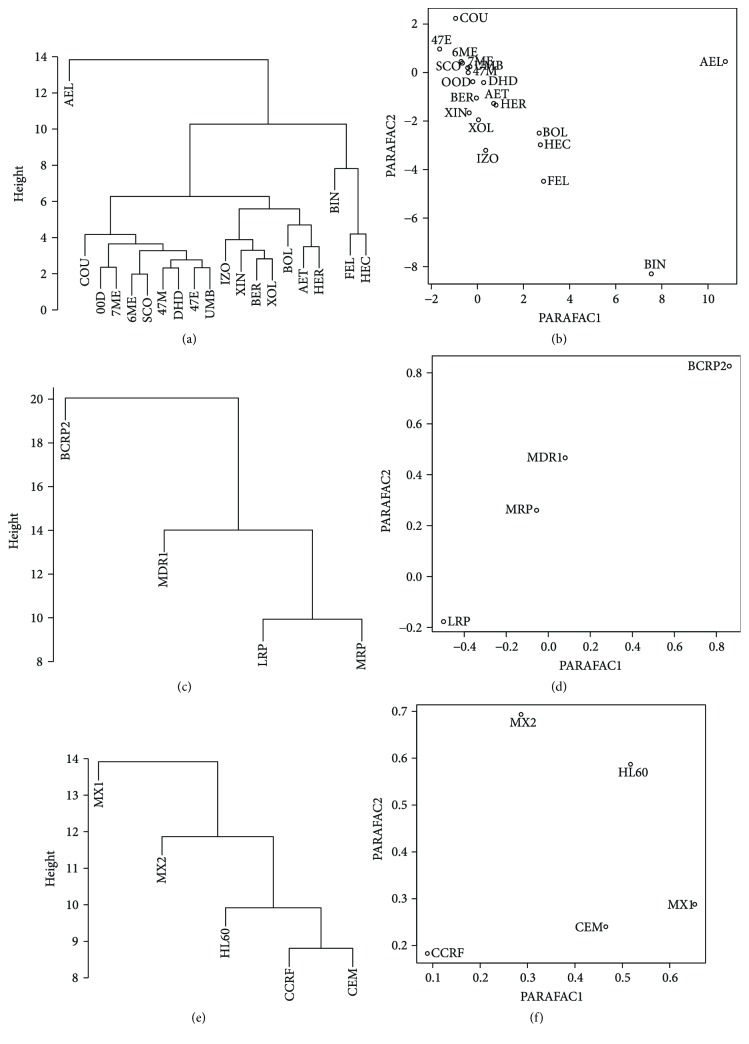
Similarity between furanocoumarin compounds (a, b), genes (c, d) and cell lines (e, f) analyzed by cluster analysis with Euclidean distance (a, c, e) and PARAFAC (b, d, f) while the mitoxantrone action.

The results of the cluster analysis were presented as dendrograms (Figures 1 and 2). As the investigated data were continuous variables, Euclidean distance was chosen as an appropriate similarity measure.

The data was organized as a tensor of dimensions: four genes (*MDR1*, *MRP*, *LRP*, and *BCRP2*), five cell lines (HL-60, HL-60/X1, HL-60/MX2, CEM/C1, and CCRF/CEM), and 8 furanocoumarin derivatives: isopimpinellin (ISO), bergapten (BER), xanthotoxol (XOL), xanthotoxin (XIN), byakangelicin (BIN), byakangelicol (BOL), heraclenin (HEC), phellopterin (FEL), and 12 simple coumarins derivatives: herniarine (HER), aesculetin (AET), dihydrocoumarin (DHD), coumarin (COU), aesculin (AEL), umbelliferone (UMB), 4-methylo-7-methoxycoumarin (4,7M), 4-methylo-7-ethoxycoumarin (4,7E), 7-methylocoumarin (7ME), 6-methylocoumarin (6ME), 0,0-dimethylofraxetin (OOD), and scoparone (SCO).

### 3.3. Coumarin Derivative Dataset

#### 3.3.1. Analysis of Similarity of the Investigated Coumarin Derivatives on the Level of Gene Expression

The cutoff height of the dendrogram (Figure 1(a)) was set to 12. Two visible clusters were observed. The first consists of furanocoumarins: isopimpinellin (ISO), bergapten (BER), xanthotoxol (XOL), xanthotoxin (XIN), byakangelicin (BIN), byakangelicol (BOL), heraclenin (HEC), and phellopterin (FEL); the second consists of coumarins: herniarine (HER), aesculetin (AET), dihydrocoumarin (DIH), coumarin (COU), aesculin (AEL), umbelliferone (UMB), 4-methylo-7-methoxycoumarin (4,7M), 4-methylo-7-ethoxycoumarin (4,7E), 7-methylocoumarin (7ME), 6-methylocoumarin (6ME), 0,0-dimethylofraxetin (OOD), and Scoparone (SCO).

The PARAFAC analysis of aforementioned data tensor explained 61.56% with an optimal number of two factors. The results grouped in two clusters. First, as previously, contains furanocoumarin derivatives, whereas the second contains the other coumarins (Figure 1(b)).

#### 3.3.2. Analysis of Similarity of Genes Expression in Cell Lines Stimulated with Coumarin Derivatives

Optimal cutoff height for clustering was chosen to be 14 (Figure 1(c)). Genes are divided into two distinct clusters: *LRP + MRP* and *BCRP2 + MDR1*. PARAFAC results (Figure 1(d)) do not show any clustering tendency.

#### 3.3.3. Analysis of Similarity of Cell Lines Specific Gene Expression Stimulated with Coumarin Derivatives

The optimal cutoff height was set to 20 (Figure 1(e)). First cluster is created by HL-60, HL-60/MX2, and HL-60/MX1 lines, not strictly similar. The rest of lines are much more similar to themselves. In PARAFAC results (Figure 1(f)), the HL-60/MX2 cell line is a visible outlier and the other lines are much more similar to themselves.

### 3.4. Coumarin Derivatives + Mitoxantrone Dataset

#### 3.4.1. Analysis of Similarity of the Investigated Coumarin Derivatives on the Level of Gene Expression—Mitoxantrone Exposed Cells

Aesculin (AEL) was found to be an outlier during cluster analysis (Figure 2(a)). Phellopterin (FEL) and heraclenin (HEC) were similar to themselves but were slightly different than byakangelicin (BIN). Umbelliferone (UMB), 0,0-dimethylofraxetin (OOD), scoparone (SCO), 7-methylocoumarin (7ME), 6-methylocoumarin (6ME), 4-methylo-7-methoxycoumarin (4,7M), 4-methylo-7-ethoxycoumarin (4,7E), and dihydrocoumarin (DIH) formed visible cluster; however, they differ to coumarine (COU).

In the next cluster, a strong similarity was found between bergaptene (BER) and xanthotoxol (XOL), which were different than xanthotoxin (XIN) and isopimpinellin (ISO).

Two-factor PARAFAC decomposition explained 45.89% of whole data (Figure 2(b)). Aesculin (AEL) and byakangelicin (BIN) were identified as outliers during this analysis.

#### 3.4.2. Analysis of Similarity of Cell Line-Specific Gene Expression Stimulated with Coumarin Derivatives and Mitoxantrone

Expression levels of *LRP* and *MRP1* genes were found to be similar (Figure 2(c)), whereas the other genes are not clustered. No clustering tendency was also observed with PARAFAC (Figure 2(d)).

### 3.5. Cell Line Similarity

CCRF/CEM and CEM/C1 cell lines were found to be most similar (Figure 2(e)), whereas the other cell lines are not clustered. No visible clustering tendency is observed with PARAFAC (Figure 2(f)).

#### 3.5.1. Changes in Gene Expression of BCRP2, LRP, MDR1, and MRP in Cell Lines after 24 h Exposition on Coumarine Derivatives (Figure 3(a))

We observed a decrease in *BCRP* gene expression to minimum −3.392 (MX2/HL-60; 4,7M; IC50) and increased gene expression to a maximum *LRP* to 2.005-fold (MX1/HL-60; OOD; IC50), *MDR1* to 2.761-fold (MX1/HL-60; 4,7M; IC50), *MRP1* to 1.407-fold (CCRF/CEM; OOD; IC50).

The mean expression levels of genes were *BCRP* −0.2346 (SD 1.06), *LRP* 0.933 (SD 0.50), *MDR1* 0.3664 (SD 1.56), and *MRP* 0.204 (SD 0.41).

#### 3.5.2. Changes in Gene Expression of BCRP, LRP, MDR1, and MRP1 in Cell Lines after 24 h Exposition on Coumarine Derivatives with Mitoxantrone (Figure 3(b))

We observed increased gene expression to a maximum *BCRP* to 3.652-fold (CEM/C1; AEL; IC50), *LRP* to 1.790-fold (MX2/HL-60; 7ME; IC50), *MDR1* to 1.973-fold (MX1/HL-60; 6ME; IC50) and a decrease of *MRP1* gene expression to a minimum −0.822 (MX1/HL-60; HER; IC50).

The mean expression levels of genes were *BCRP* 0.234 (SD 0.92), *LRP* 0.406 (SD 0.82), *MDR1* 0.278 (SD 0.53), and *MRP1*-0.006 (SD 0.26).

#### 3.5.3. Changes in Gene Expression of BCRP, LRP, MDR1, and MRP1 in Cell Lines after 24 h Exposition on Furanocoumarin Derivatives (Figure 3(c))

We observed an increase of gene expression to a maximum *BCRP* to 2.850-fold (MX1/HL-60; FEL; IC50), *LRP* to 1.358-fold (MX1/HL-60; BOL; IC50), *MDR1* to 2.513-fold (MX2/HL-60; BOL; IC50), and *MRP* 0.841-fold (CCRF/CEM; BER; IC50).

The mean expression levels of genes were *BCRP 2*0.800 (SD 1.13), *LRP* 0.647 (SD 0.37), *MDR1* 0.896 (SD 0.83), and *MRP1* −0.189 (SD 0.51).

#### 3.5.4. Changes in Gene Expression of BCRP, LRP, MDR1, and MRP1 in Cell Lines after 24 h Exposition on Furanocoumarin Derivatives with Mitoxantrone (Figure 3(d))

We observed decreased gene expression to a minimum *BCRP* to −1.6571-fold (MX1/HL-60; IZO; IC50), *LRP* to −1.176-fold (CEM/C1; BIN; IC50), and *MRP1* to −1.213-fold (CEM/C1; BOL; IC50) and increase of *MDR1* gene expression to a maximum 2.325-fold (CEM/C1; BIN; IC50).

The mean expression levels of genes were *BCRP* −0.186 (SD 0.94), *LRP* −0.012 (SD 0.48), *MDR1* 0.029 (SD 0.87), and *MRP1* −0.541 (SD 0.30).

## 4. Discussion

Multidrug resistance is one of the main causes of failure in anticancer therapy. For over 40 years, research targeted at searching for compounds that abolish the multidrug resistance effect has been conducted by many research teams all over the world. The mechanism of multidrug resistance can be explained by overexpression of membrane transporters, mainly from the ABC family, which remove drugs from the cancer cell in an active way.

The cytotoxicity of the examined coumarins was estimated using trypan blue vital staining in the presence of M(+) and absence of mitoxantrone M. The IC10, IC10M(+), IC50, and IC50 M(+) values were determined. The cells of the five cancer cell lines exposed to coumarin derivatives presented diverse cytotoxicity dependent on the dose of IC10, IC10M(+), IC50, and IC50M(+).

Received dose values the IC10, IC10M(+), IC50, and IC50M(+) of coumarin compounds show high cytotoxicity for all tested cell lines. IC50 doses of coumarin compounds were lower than those for furanocoumarins, indicating more toxic effects on tumor cells. A similar conclusion was made by Yang et al. [35], in which ostol—a representative of simple coumarin—showed much higher cytotoxicity than the furanocoumarins investigated.

Against the background of the results, it can be concluded that lines without resistance phenotype are more susceptible to the effects of coumarin compounds. The least sensitive cell line is HL-60/MX1 and HL-60/MX2 derived from promyelocytic leukemia.

Coumarin substances, both natural and synthetic, are often screened for cancer toxicity in various cancer cell lines [36–39]. A literature review shows that the most commonly studied leukemia is HL-60.

Yang et al. [35] isolated five coumarin substances from *Cnidium monnieri* L. fruits and then examined their toxicity towards HL-60 cells. IC50 values have been established on a level not more than 50 *μ*M for isopimpinellin (ISO), bergapten (BER), and xanthotoxin (XIN). Similar values were obtained in our work for isopimpinellin (ISO) and bergapten (BER) but were slightly higher for xanthotoxin (XIN) 61 *μ*M/ml.

Cluster analysis with the Euclidean distance measure based on the MDR gene expression divided the examined compounds into two groups. The first group comprises furanocoumarin derivatives and the second one comprises coumarin derivatives. Such a division shows that these compounds have different mechanisms of action on the transcriptome of cancer cells.

Most of the investigators in the work of coumarin compounds induce increased expression of *MDR1*, *BCRP*, *LRP*, and *MRP* genes in leukemia cells [40–42]. This phenomenon can be explained by the correct detection by defense mechanisms of cells in response to coumarin compounds, which are recognized by the cell as xenobiotics.

However, to our knowledge, this is the first report describing the study of the effect of coumarin compounds with mitoxantrone on the expression of multidrug resistance genes. Our research show, in the case of furanocoumarin compounds in the presence of mitoxantrone, the expression of the *MDR1*, *BCRP*, *LRP*, and *MRP* genes was reduced, which may be of interest in a therapeutic context.

Studying the level of gene expression in the ABC family is often used in clinical practice. The levels of gene expression in the ABC family are examined in patients prior to initiation of treatment. The result is determined by further therapy. Evaluation of ABC gene expression in leukemia diagnostics may contribute to the early identification of patients at risk for treatment failure who require individual therapy [40, 41, 43].

On the basis of the results of the analysis of the ABC family gene expression in leukemia cells exposed to the examined compounds and statistical analysis, it is concluded that the furanocoumarin compounds are more promising in terms of their mechanism of action.

The high activity of coumarin compounds seems to be a basis for the design of new analogues characterized by increased activity and safety of use. The challenge for researchers is to create new drugs based on the design and synthesis of highly active derivatives and the elucidation of their mechanism of action. Recent advances in the design of new union structures may lead to the discovery of novel anticancer drugs. Increased cancer mortality and high medical costs are the incentive to continually seek for anticancer drugs with increased efficacy.

The obtained results significantly broaden the knowledge about the anticancer effects of coumarin compounds and their effect on the expression of *MDR1*, *BCRP*, *LRP*, and *MRP* multidrug induction genes of tumor cells derived from the human hematopoietic system: CEM/C1, CCRF/CEM, HL-60, HL-60/MX1, and HL-60/MX2.

Overexpression of resistance genes, resulting in cell-induced drug resistance, is of great importance in the treatment of cancer. Often, it is a major factor in the failure of therapy. Therefore, it is important to look for new compounds that will safely modulate the expression of genes that affect multidrug resistance.

## 5. Conclusions

1. For a majority of the coumarin compounds, the IC10, IC10M(+), IC50, and IC50M(+) values were estimated for the first time. The values obtained show high cytotoxicity to the examined cell lines, that is, CEM/C1, CCRF/CEM, HL-60, HL-60/MX1, and HL-60/MX2.
2. It was observed that cell lines without the resistance phenotype are more sensitive to the coumarin compounds. HL-60/MX1 and HL-60/MX2 cell lines derived from promyelocytic leukemia are the least sensitive.
3. In the case of furanocoumarin compounds, in the presence of mitoxantrone, the expression of the *MDR1*, *BCRP*, *LRP*, and *MRP* genes was reduced, which may be of interest in a therapeutic context.
4. Cluster analysis conducted based on gene expression clearly divided the examined compounds into two groups. The first group comprises furanocoumarin derivatives, and the second group includes coumarin derivatives. Such a division shows that these compounds have different mechanisms of action on the transcriptome of cancer cells. The PARAFAC analysis confirms this observation.
5. The obtained results significantly broaden the knowledge about the anticancer effects of coumarin compounds and their effect on the expression of *MDR1*, *BCRP*, *LRP*, and *MRP* genes in tumor cell lines derived from human hematopoietic system: CEM/C1, CCRF/CEM, HL-60, HL-60/MX1, and HL-60/MX2.

## Figures and Tables

**Figure 3:**
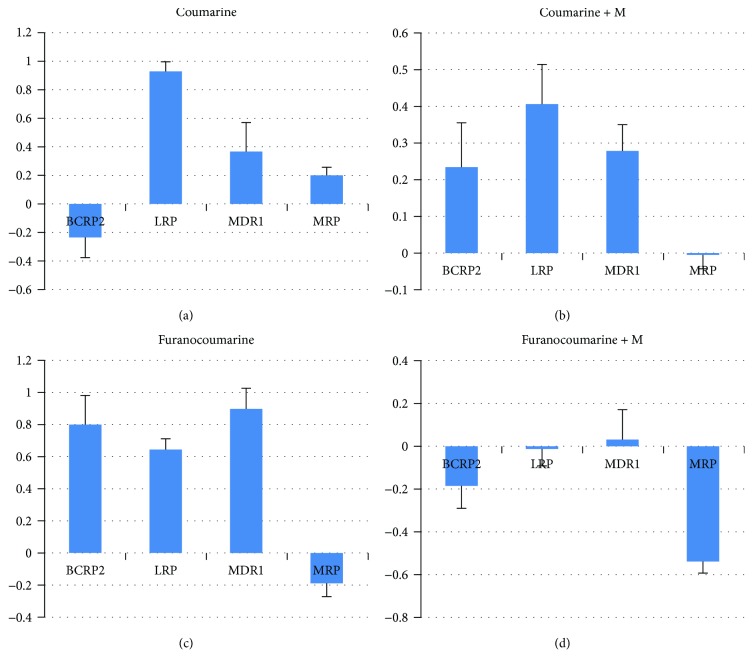
The mean *BCRP*, *LRP*, *MDR1*, and *MRP1* gene expression level in the cell lines CCRF/CEM, CEM/C1, HL-60, HL-60/MX1, and HL-60/MX2 after 24 h exposition on: coumarin (a), coumarin with mitoxantrone (b), furanocoumarins (c), and furanocoumarins without mitoxantrone (d) derivatives.

**Table 1:** IC10 values for line cells CEM/C1, CCRF/CEM, HL-60, HL-60/MX1, and HL-60/MX2 (*μ*M). SD: standard deviation.

	CEM/C1	CCRF/CEM	HL-60	HL-60/MX1	HL-60/MX2
	IC10 ± SD	IC10 ± SD	IC10 ± SD	IC10 ± SD	IC10 ± SD
ISO	13.0 ± 2.5	6.9 ± 1.0	4.6 ± 0.6	13.8 ± 2.2	16.4 ± 2.1
BER	12.1 ± 2.0	8.8 ± 1.7	4.6 ± 1.1	15.8 ± 2.6	11.4 ± 2.4
XOL	11.7 ± 3.1	7.6 ± 1.0	4.6 ± 1.1	13.4 ± 2.0	11.1 ± 2.5
XIN	12.8 ± 2.1	5.7 ± 3.6	9.3 ± 3.1	11.7 ± 2.6	10.5 ± 2.0
BIN	15.5 ± 2.6	8.5 ± 3.1	4.6 ± 2.6	3.0 ± 2.5	12.9 ± 1.7
BOL	15.5 ± 2.5	6.4 ± 2.0	9.2 ± 2.5	11.5 ± 2.5	10.5 ± 1.0
HEC	12.7 ± 2.5	6.0 ± 3.1	9.2 ± 2.0	15.0 ± 3.2	18.2 ± 2.0
FEL	21.4 ± 3.5	5.9 ± 0.4	9.8 ± 3.1	14.5 ± 2.8	16.4 ± 2.0
HER	4.4 ± 1.2	4.3 ± 1.2	3.4 ± 1.9	18.2 ± 2.5	5.9 ± 0.9
AET	4.5 ± 0.8	3.6 ± 2.2	9.0 ± 3.1	11.1 ± 4.4	5.9 ± 1.5
DHD	5.3 ± 0.6	5.0 ± 1.5	4.8 ± 1.0	4.0 ± 4.3	5.9 ± 1.1
COU	5.3 ± 0.5	1.0 ± 3.1	5.0 ± 1.5	4.0 ± 4.2	4.3 ± 2.0
AEL	4.2 ± 1.1	4.2 ± 0.5	5.9 ± 4.2	2.5 ± 3.5	8.3 ± 1.1
UMB	5.0 ± 0.6	4.0 ± 1.1	5.5 ± 1.5	13.3 ± 2.2	8.3 ± 1.0
4,7M	3.6 ± 0.2	5.5 ± 0.5	2.3 ± 1.1	16.7 ± 2.8	6.7 ± 1.5
4,7E	1.9 ± 1.0	4.5 ± 0.8	1.7 ± 1.6	16.7 ± 2.6	7.7 ± 1.4
7ME	2.8 ± 1.0	3.6 ± 1.1	7.7 ± 2.1	14.3 ± 4.2	8.3 ± 1.6
6ME	2.6 ± 1.0	5.9 ± 2.1	6.2 ± 2.0	18.2 ± 3.8	5.5 ± 1.6
0,0D	2.6 ± 0.3	4.3 ± 1.4	2.9 ± 0.8	11.1 ± 3.4	8.3 ± 2.2
SCO	2.7 ± 0.5	6.2 ± 0.7	3.6 ± 0.3	22.2 ± 3.2	7.1 ± 1.9

**Table 2:** IC10 + M values for line cells CEM/C1, CCRF/CEM, HL-60, HL-60/MX1, and HL-60/MX2 (*μ*M). SD: standard deviation.

	CEM/C1	CCRF/CEM	HL-60	HL-60/MX1	HL-60/MX2
	IC10 ± SD	IC10 ± SD	IC10 ± SD	IC10 ± SD	IC10 ± SD
ISO	12.8 ± 2.5	8.0 ± 0.5	5.6 ± 0.5	14.0 ± 1.5	16.8 ± 2.5
BER	12.3 ± 2.0	7.7 ± 1.0	5.2 ± 0.4	13.4 ± 1.8	12.7 ± 2.0
XOL	13.0 ± 2.1	9.1 ± 1.1	5.6 ± 0.6	10.3 13.1	10.7 ± 1.4
XIN	14.7 ± 2.1	10.2 ± 2.1	9.4 ± 1.1	12.0 ± 2.1	10.2 ± 1.3
BIN	15.0 ± 2.6	8.2 ± 1.6	6.6 ± 0.6	11.8 ± 2.6	11.1 ± 2.6
BOL	12.0 ± 2.5	7.1 ± 0.8	14.7 ± 2.5	13.0 ± 2.5	11.0 ± 2.5
HEC	14.3 ± 2.5	6.6 ± 2.1	10.2 ± 2.5	14.0 ± 2.5	15.9 ± 2.5
FEL	17.3 ± 3.5	6.4 ± 2.6	11.0 ± 3.5	12.2 ± 3.5	15.7 ± 3.5
HER	4.2 ± 0.6	5.1 ± 0.5	4.2 ± 0.4	17.3 ± 2.5	6.4 ± 0.5
AET	5.3 ± 0.5	4.3 ± 1.0	10.0 ± 2.0	10.2 ± 2.0	6.2 ± 1.0
DHD	5.1 ± 0.6	5.6 ± 1.1	5.8 ± 0.7	3.8 ± 0.5	6.2 ± 1.1
COU	5.0 ± 0.4	1.2 ± 0.1	6.2 ± 0.5	4.2 ± 0.5	4.8 ± 0.6
AEL	4.8 ± 0.6	4.0 ± 0.6	6.4 ± 2.6	2.3 ± 2.6	7.6 ± 2.6
UMB	5.2 ± 0.5	4.2 ± 0.5	6.8 ± 2.5	13.0 ± 2.5	7.8 ± 1.5
4,7M	3.2 ± 0.5	6.3 0.7	4.1 ± 2.5	14.8 ± 2.5	6.2 ± 0.5
4,7E	1.7 ± 0.2	5.2 ± 0.5	2.3 ± 3.5	14.8 ± 3.5	7.2 ± 1.5
7ME	4.1 ± 0.5	4.2 ± 0.5	8.2 ± 2.5	13.8 ± 2.5	7.6 ± 1.5
6ME	3.2 ± 0.8	6.2 ± 1.0	6.8 ± 2.0	16.2 ± 2.0	4.8 ± 2.0
0,0D	2.2 ± 0.6	4.8 ± 1.1	4.0 ± 0.5	10.4 ± 2.1	7.4 ± 1.1
SCO	2.4 ± 0.2	6.8 ± 2.1	4.2 ± 2.1	20.2 ± 2.1	6.4 ± 2.1

**Table 3:** IC50 values for line cells CEM/C1, CCRF/CEM, HL-60, HL-60/MX1, and HL-60/MX2 (*μ*M). SD: standard deviation.

	CEM/C1	CCRF/CEM	HL-60	HL-60/MX1	HL-60/MX2
	IC50 ± SD	IC50 ± SD	IC50 ± SD	IC50 ± SD	IC50 ± SD
ISO	21.5 ± 4.5	10.0 ± 4.2	21.5 ± 2.5	21.0 ± 4.2	26.0 ± 5.7
BER	28.5 ± 7.5	15.5 ± 4.5	16.5 ± 3.6	16.0 ± 4.9	36.5 ± 3.6
XOL	15.5 ± 4.5	12.5 ± 4.5	28.0 ± 5.0	19.0 ± 6.1	45.0 ± 9.0
XIN	24.0 ± 5.0	23.0 ± 7.6	61.0 ± 7..0	36.0 ± 5.1	46.5 ± 5.5
BIN	13.0 ± 4.5	5.5 ± 4.5	22.0 ± 3.6	8.0 ± 1.0	29.0 ± 4.0
BOL	19.0 ± 4.0	15.5 ± 4.0	43.0 ± 6.1	19.5 ± 2.6	34.5 ± 5.5
HEC	22.0 ± 5.3	18.0 ± 4.2	45.0 ± 10.1	18.0 ± 6.4	29.5 ± 5.0
FEL	8.0 ± 4.0	15.5 ± 4.5	42.0 ± 5.0	31.0 ± 8.0	40.5 ± 4.5
HER	25.0 ± 4.0	25.0 ± 4.0	25.0 ± 4.6	56.6 ± 4.1	30.0 ± 4.0
AET	25.0 ± 3.5	20.0 ± 2.6	25.0 ± 3.6	67.6 ± 7.7	30.0 ± 6.5
DHD	25.0 ± 4.0	20.0 ± 2.5	20.0 ± 3.2	47.2 ± 5.5	30.0 ± 4.6
COU	25.0 ± 5.0	30.0 ± 4.7	20.0 ± 3.0	43.8 ± 4.9	40.0 ± 4.9
AEL	25.0 ± 6.6	25.0 ± 3.5	20.0 ± 4.2	47.2 ± 4.6	40.0 ± 6.2
UMB	25.0 ± 3.2	25.0 ± 3.3	20.0 ± 2.1	42.8 ± 4.9	40.0 ± 4.0
4,7M	30.0 ± 3.8	20.0 ± 3.6	20.0 ± 4.2	30.0 ± 3.5	30.0 ± 5.3
4,7E	10.0 ± 1.5	25.0 ± 4.6	10.0 ± 2.5	32.4 ± 3.1	40.0 ± 3.8
7ME	15.0 ± 2.6	25.0 ± 3.1	25.0 ± 4.5	36.8 ± 3.1	40.0 ± 6.2
6ME	30.0 ± 5.7	30.0 ± 4.9	25.0 ± 2.1	39.5 ± 6.7	30.0 ± 4.3
0,0D	25.0 ± 3.5	30.0 ± 3.6	25.0 ± 4.0	35.7 ± 4.2	40.0 ± 5.5
SCO	20.0 ± 4.2	30.0 ± 4.6	25.0 ± 3.5	42.8 ± 4.8	40.0 ± 3.5

**Table 4:** IC50 + M values for line cells CEM/C1, CCRF/CEM, HL-60, HL-60/MX1, and HL-60/MX2 (*μ*M). SD: standard deviation. ^∗^The survival of line cells after exposure to compounds at 50 *μ*mol concentration drops to about 20%.

	CEM/C1	CCRF/CEM	HL-60	HL-60/MX1	HL-60/MX2
	IC50 ± SD	IC50 ± SD	IC50 ± SD	IC50 ± SD	IC50 ± SD
ISO	15.5 ± 4.5	10.5 ± 4.2	29.0 ± 2.5	28.0 ± 4.2	28.5 ± 5.7
BER	15.5 ± 7.5	14.0 ± 4.5	18.5 ± 3.6	24.0 ± 4.9	23.0 ± 3.6
XOL	15.5 ± 4.5	12.0 ± 4.5	42.0 ± 5.0	28.0 ± 6.1	37.5 ± 9.0
XIN	25.0 ± 5.0	16.0 ± 7.6	60.5 ± 10.0	31.0 ± 5.1	37.5 ± 5.5
BIN	13.0 ± 4.5	11.0 ± 4.5	27.0 ± 3.6	25.5 ± 4.0	23.0 ± 6.0
BOL	12.0 ± 4.0	^∗^	19.0 ± 6.1	17.0 ± 2.6	30.0 ± 5.5
HEC	28.0 ± 5.3	^∗^	46.5 ± 10.1	16.0 ± 6.4	36.0 ± 5.0
FEL	10.5 ± 4.0	^∗^	36.0 ± 5.0	18.0 ± 8.0	42.0 ± 4.5
HER	23.2 ± 4.5	28.2 ± 4.5	28.1 ± 4.5	61.0 ± 4.5	28.2 ± 4.5
AET	23.6 ± 7.5	24.1 ± 7.5	22.6 ± 7.5	68.4 ± 7.5	29.4 ± 7.5
DHD	23.6 ± 4.5	23.2 ± 4.5	22.8 ± 4.5	52.3 ± 4.5	28.6 ± 4.5
COU	23.2 ± 5.0	34.0 ± 5.0	23.1 ± 5.0	47.8 ± 5.0	38.2 ± 5.0
AEL	24.0 ± 4.5	22.4 ± 4.5	21.4 ± 4.5	49.2 ± 4.5	38.8 ± 4.5
UMB	23.8 ± 4.0	22.6 ± 4.0	21.2 ± 4.0	44.8 ± 4.0	38.6 ± 4.0
4,7M	26.2 ± 5.3	23.6 ± 5.3	8.2 ± 5.3	34.0 ± 5.3	32.4 ± 5.3
4,7E	12.2 ± 4.0	27.6 ± 4.0	28.2 ± 4.0	34.2 ± 4.0	41.8 ± 4.0
7ME	13.8 ± 4.5	27.2 ± 4.5	27.6 ± 4.5	40.1 ± 4.5	37.6 ± 4.5
6ME	26.4 ± 7.5	34.0 ± 7.5	26.4 ± 7.5	42.0 ± 7.5	28.2 ± 7.5
0,0D	22.3 ± 4.5	32.2 ± 4.5	27.2 ± 4.5	38.2 ± 4.5	37.2 ± 4.5
SCO	18.7 ± 5.0	31.4 ± 5.0	27.4 ± 5.0	44.6 ± 5.0	36.4 ± 5.0

## Abbreviations

HL-60: Human Caucasian promyelocytic leukemia from the American Type Culture Collection (ATCC CCL-240™)
HL-60/MX1: Human Caucasian acute promyelocytic leukemia from the American Type Culture Collection (ATCC CRL-2258™)
HL-60/MX2: Human Caucasian acute promyelocytic leukemia from the American Type Culture Collection (ATCC CRL-2257™)
CEM/C1: Human Caucasian acute lymphoblastic leukemia from the American Type Culture Collection (ATCC CRL-2265™)
CCRF/CEM: Human Caucasian acute lymphoblastic leukemia from the American Type Culture Collection (ATCC CCL-119™)
ISO: Isopimpinellin
BER: Bergapten
XOL: Xanthotoxol
XIN: Xanthotoxin
BIN: Byakangelicin
BOL: Byakangelicol
HEC: Heraclenin
FEL: Phellopterin
HER: Herniarine
AET: Aesculetin
DHD: Dihydrocoumarin
COU: Coumarin
AEL: Aesculin
UMB: Umbelliferone
4,7M: 4-methylo-7-methoxycoumarin
4,7E: 4-methylo-7-ethoxycoumarin
7ME: 7-methylocoumarin
6ME: 6-methylocoumarin
OOD: 0,0-dimethylofraxetin
SCO: Scoparone.

## Supplementary Materials

Supplementary Materials Amplification plot.
