# Supplementary material for: Modulation of Multidrug Resistance Gene Expression by Coumarin Derivatives in Human Leukemic Cells
Source: Oxid Med Cell Longev. 2017 Dec 13;2017:5647281. doi: 10.1155/2017/5647281 (PMC5745744; doi:10.1155/2017/5647281)
Supplement: Supplementary Materials — Amplification plot. [file 5647281.f1.pdf]

Amplification Plot

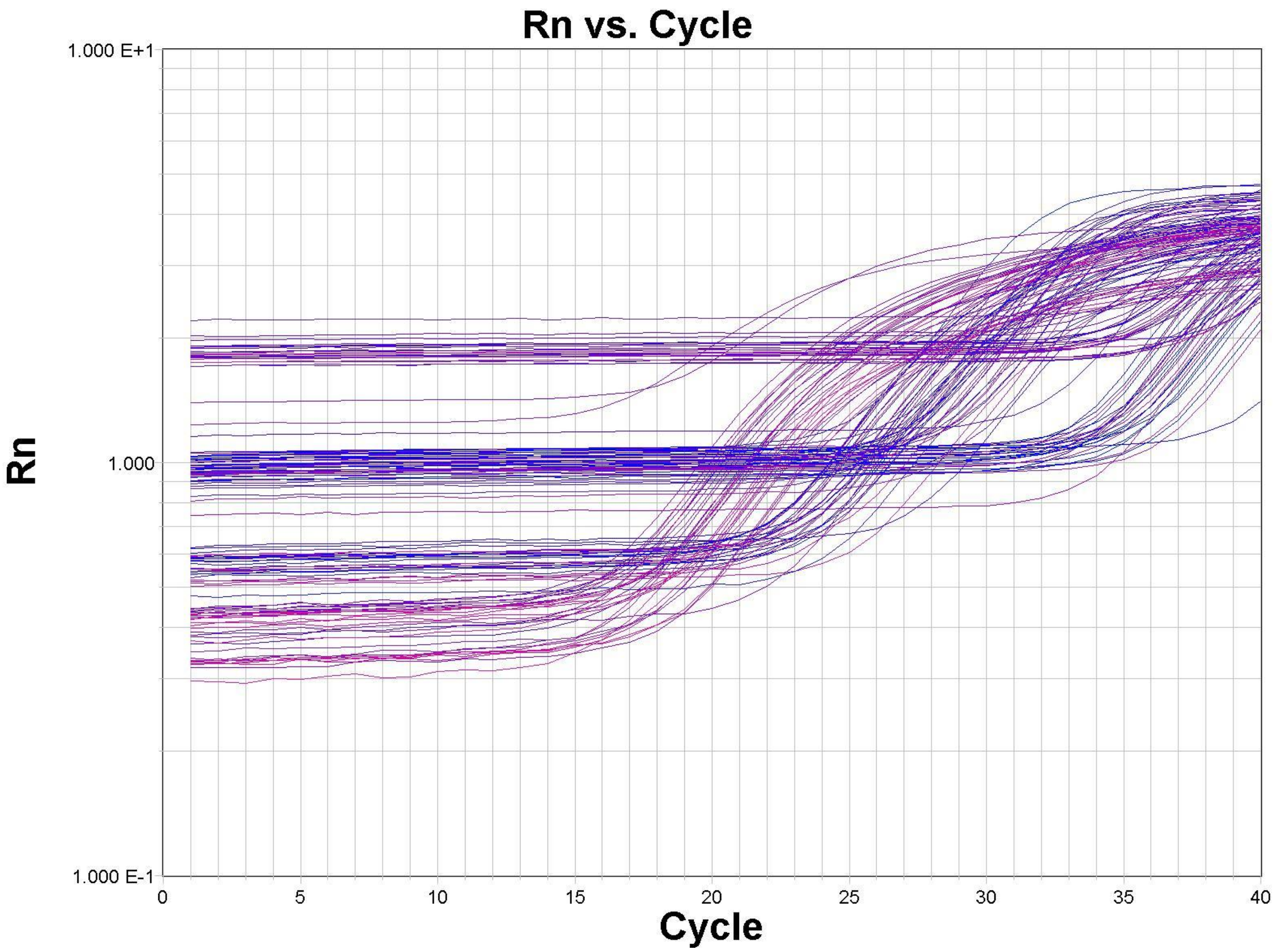

Coumarin+M, CEM/C1

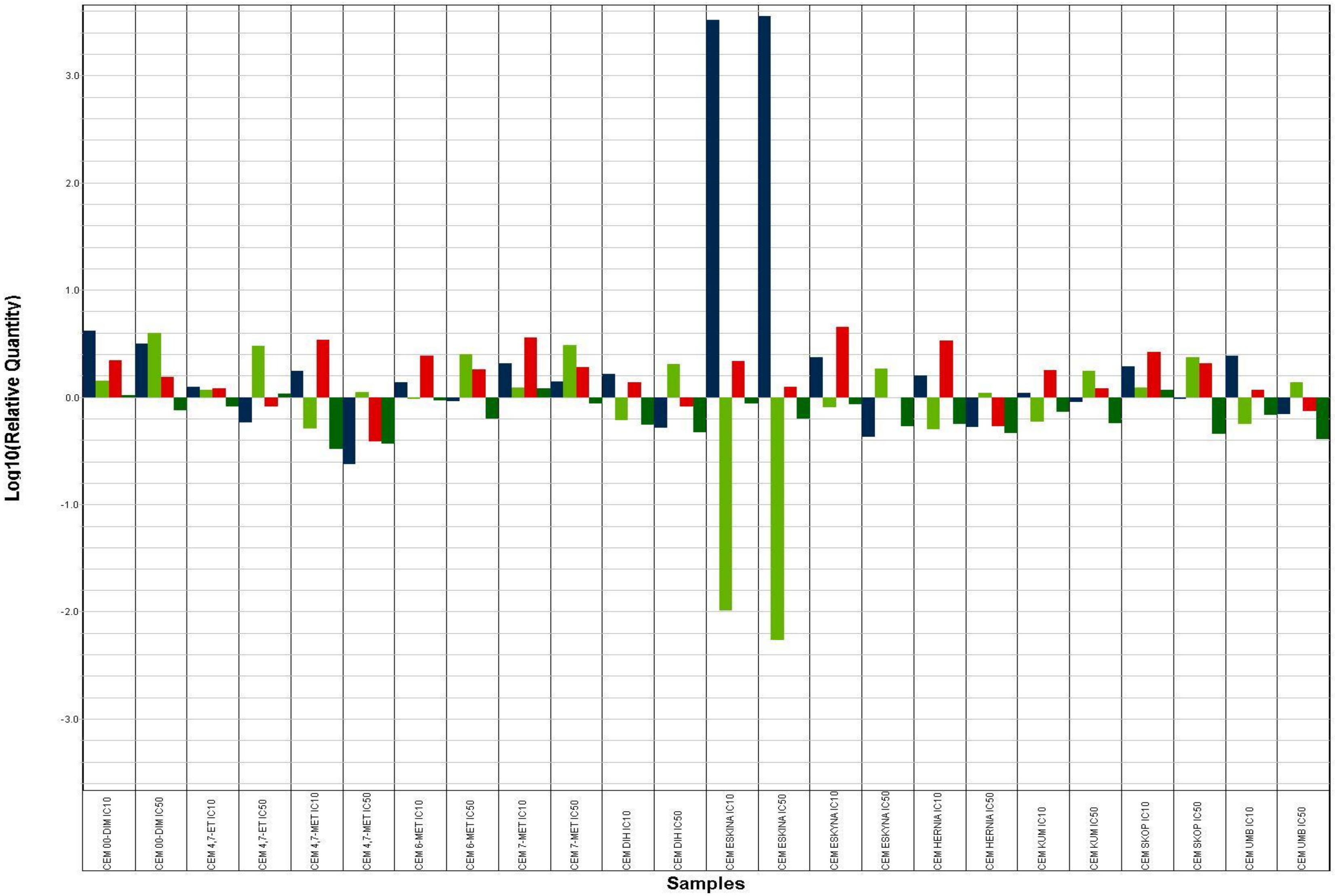

Coumarin+M, CEM/C1

Study Name	: Coumarin + M, CEM/C1.sdm
Software Version	: RQ Manager 1.2
Number of Plates in Study	: 9
User	:
Comments	:
